# Retreatment Predictions in Odontology by means of CBR Systems

**DOI:** 10.1155/2016/7485250

**Published:** 2016-01-14

**Authors:** Livia Campo, Ignacio J. Aliaga, Juan F. De Paz, Alvaro Enrique García, Javier Bajo, Gabriel Villarubia, Juan M. Corchado

**Affiliations:** ^1^Department of Estomatology II, Complutense University of Madrid, Plaza Ramón y Cajal, s/n, 28040 Madrid, Spain; ^2^Biomedical Research Institute of Salamanca/BISITE Research Group, Calle Espejo, s/n, 37007 Salamanca, Spain; ^3^Departamento de Inteligencia Artificial, Universidad Politécnica de Madrid, Campus Montegancedo, Boadilla del Monte, 28660 Madrid, Spain; ^4^Osaka Institute of Technology, Osaka 535-8585, Japan

## Abstract

The field of odontology requires an appropriate adjustment of treatments according to the circumstances of each patient. A follow-up treatment for a patient experiencing problems from a previous procedure such as endodontic therapy, for example, may not necessarily preclude the possibility of extraction. It is therefore necessary to investigate new solutions aimed at analyzing data and, with regard to the given values, determine whether dental retreatment is required. In this work, we present a decision support system which applies the case-based reasoning (CBR) paradigm, specifically designed to predict the practicality of performing or not performing a retreatment. Thus, the system uses previous experiences to provide new predictions, which is completely innovative in the field of odontology. The proposed prediction technique includes an innovative combination of methods that minimizes false negatives to the greatest possible extent. False negatives refer to a prediction favoring a retreatment when in fact it would be ineffective. The combination of methods is performed by applying an optimization problem to reduce incorrect classifications and takes into account different parameters, such as precision, recall, and statistical probabilities. The proposed system was tested in a real environment and the results obtained are promising.

## 1. Introduction

Bioinformatics can be applied to various fields of medicine, although it is normally used in fields associated with genetic expressions [1, 2], protein analysis [3], sequencing [4], and so forth. Its use is not as commonly applied to more restricted levels such as private medical consultations. Nevertheless, while bioinformatics does fall within this scope, medical doctors often use what is referred to as knowledge extraction, which is based on experience gained over time from experts in the field. Expert knowledge is composed of the prediction or classification of pathologies in relation to a set of symptoms exhibited by the patient. The decisions made by odontologists have been traditionally based on past experiences of previous treatment cases. There are normally too many variables to consider, which has in fact resulted in the high failure rate of retreatments and the inability to easily create expert knowledge, particularly from doctors recently new to the profession. Consequently, it is necessary to provide new solutions that facilitate the decision-making process of odontologists and can lead to decisions that minimize the failure of endodontic treatments and retreatments. Decision support systems can notably help odontologists make decisions, and case-based reasoning is especially appropriate for this kind of problems.

In odontology, the success rate of endodontic therapy is 90%, which leaves a failure rate of 10%. Thus, an odontologist would greatly appreciate the ability to use artificial intelligence techniques to analyze the cases falling within this 10% and determine whether retreatment or extraction is preferable. The problem in 40% of these cases is the result of root crown fractures, which in turn represent 5% of all dental fractures. The bacterial recolonization of the root canal and the subsequent appearance of radiological symptoms represent 15% of endodontic failure [5–7]. It is not possible to find many published studies within the field of bioinformatics that address the problem previously presented. Existing works are limited to statistical analysis, which extracts the variables that are differentiated in different groups of patients and, according to the results obtained, make it possible to characterize relevant variables. However, this method does not permit the simultaneous analysis of the influence of the different variables. Statistical analysis is limited to the application of specific tests such as chi-square [8], Mann-Whitney [9], or Kruskal-Wallis test [10]. Nevertheless, it is necessary to create a new process that can combine all the information gathered in an intelligent way in order to perform a final classification and prediction that can help the odontologists to make more precise decisions.

This work proposes an innovative reasoning system to predict the success of retreatments. The proposed reasoning system uses past experiences to propose new solutions. CBR (case-based reasoning) systems execute a CBR cycle composed of 4 stages: retrieve (to recover past experiences), reuse (to obtain a new solution based on the retrieved past experiences), revise (to evaluate the obtained solution), and retain (to learn from the new experience). The CBR system proposed in this paper recovers a set of variables for a group of patients. This dataset is used as an input for the reuse phase of the CBR system. The reuse phase incorporates new classification techniques during the reuse phase, not previously used for this kind of problem, in order to generate a classification for the new patient. During the reuse phase, the CBR system incorporates classifiers based on Bayesian networks. The combination of both methods is achieved by applying an optimization problem in which the functional objective is defined in order to reduce false negatives (not advising retreatment when it is in fact advisable). Traditional statistical techniques are applied during the revise phase to facilitate the interpretation of the results by selecting the variables that present different characteristics from those in the groups of individuals. One of the advantages of the proposed system is that it can be adjusted to human behavior, given that they are based on the analysis of previous information in order to provide new predictions.

The remainder of the paper is structured as follows: Section 2 revises related works, focusing on prediction systems used in this kind of problem; Section 3 presents the proposed predictive mechanism, describing in detail the stages of the CBR system; Section 4 presents a case study and the results obtained; finally, Section 5 presents the conclusions obtained.

## 2. Prediction Systems

The use of predictive techniques in medicine and especially in the field of odontology has been studied since the late 1980s, at which time a statistical analysis of clinical data was the primary technique applied.

In 2001, Chugal et al. published data related to a study of teeth extracted after unsuccessful endodontic treatments at the University of Connecticut's School of Dental Medicine. The patients included in this study were treated between 1988 and 1992 in the graduate program and had experienced unsuccessful endodontic treatment within the previous four years. Variables were taken from both the clinical trial and the X-rays taken at the time of the endodontic treatment. The data obtained in this case were studied with contingency tables and the chi-squared test. The risk factors were compared using *t*-tests for independent groups, or with nonparametric tests (Mann-Whitney or Kruskal-Wallis) [11].

In 2010, using the same characteristics, Givol et al. published the results of his study performed in patients from private clinics in Israel. In this case, all the possible clinical variables prior and subsequent to the endodontic treatment were taken from 5,217 patients treated between 1992 and 2008. The data were also studied using chi-squared [12] statistical tests.

In July of 2011, Song et al. presented the data relative to a study performed on patients from the Department of Conservative Dentistry at the Dental College of Yonsei University, Seoul, Korea, between August 2004 and December 2008. Included in this study were patients who had undergone unsuccessful endodontic treatment and were in need of periapical surgery. Song took into account the clinical and X-ray data from prior to the treatment, demographic data, and data subsequent to the failed treatment. To analyze the factors that could predict the endodontic failure, he applied a chi-squared statistical study [13].

Of the previously cited works, none used artificial intelligence or case-based reasoning; nor did any use predictive tools other than the application of statistical studies to analyze risk factors. The use of this type of system offers, therefore, a wide area of study within the field of odontology and in particular with the prediction of unsuccessful endodontic treatments.

## 3. Proposed Reasoning System

The purpose of CBR is to solve new problems by adapting solutions that have been used to solve similar problems in the past [14]. The primary concept when working with CBRs is the concept of case. A case can be defined as a past experience and is composed of three elements: a problem description which describes the initial problem, a solution which provides the sequence of actions carried out in order to solve the problem, and the final state which describes the state achieved once the solution was applied. A CBR manages cases (past experiences) to solve new problems. The way cases are managed is known as the CBR cycle and consists of four sequential steps which are recalled every time a problem needs to be solved: retrieve, reuse, revise, and retain. Each of the steps of the CBR life cycle requires a model or method in order to perform its mission. The algorithms selected for the retrieval of cases should be able to search the case base and select the problem and corresponding solution most similar to the new situation. Once the most important cases have been retrieved, the reuse phase begins, in which the solutions for the retrieved cases are adapted and a new solution is generated. During this stage a mixture based on Bayesian networks is used to carry out the final classification. The revise phase consists of an expert revision for the proposed solution. Finally, the retain phase allows the system to learn from the experiences obtained in the three previous phases, consequently updating the cases memory.

**Figure 1:**
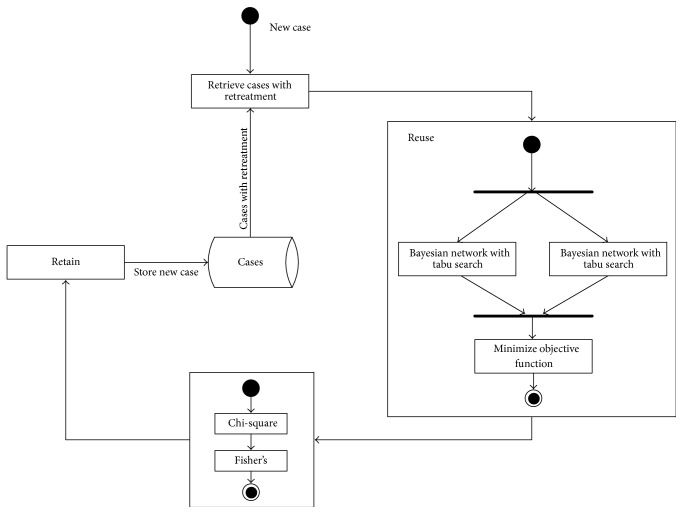
Proposed CBR system.

In this work, we propose a predictive system based on the CBR paradigm, specifically designed to be applied in the field of odontology.  Figure 1 depicts the CBR presented in this paper. As seen in Figure 1, the most innovative algorithms are included in the reuse phase, where a mixture of Bayesian networks is used. Another innovation can be observed in the revise phase, where the relevant variables are recovered by applying statistical tests to facilitate the process of reviewing the results provided during the reuse phase.

Figure 1 shows the four stages of the proposed CBR system: retrieve, reuse, revise, and retain, which are described in detail in the following subsections.

The cases are defined according to the variables and the final classification of the case; the cases are defined according to the following expression:

(1) $$\begin{array}{c} \left(\;v_{1},v_{2},\ldots ,v_{n}\;\right), \end{array}$$

where *v*_*i*_ with *i* = 1 ⋯ *n* − 1 represent the input variables and *v*_*n*_ the predicted variable with the final classification.

### 3.1. Retrieve

During the retrieve phase, existing cases in which a retreatment was performed are selected from the case memory. This eliminates all cases that involve only an initial treatment. During the reuse phase, all of the cases are selected to generate the prediction model.

### 3.2. Reuse

During the reuse phase, previously retrieved cases are selected and an associated classifier is built. The classification algorithm proposes a mixture of experts, where different methods are taken into consideration, including decision trees, decision rules, probabilistic models, fuzzy models, function-based algorithms, and ensemble. The system selects these algorithms for each kind of method: decision rules RIPPER [15], OneR [16], M5 [17], decision trees J48 [18], CART [19] (Classification and Regression Trees) [20], probabilistic models naive Bayes [21], fuzzy models *K*-NN (*K*-Nearest Neighbors) [20], Bayesian networks [22], Support Vector Machine (SVM) [23], and finally ensemble, such as Bagging [24] and Ada-Boosting [25]. In this paper, the technique selected to carry out the classification phase corresponds to a mixture of classifiers based on Bayesian networks. The mixture of classifiers minimizes a specific functional objective, which prioritizes the option for retreatment. The new case is then introduced and classified according to the classifier built in this phase. The classifiers and the mixture used in this study are explained in the following subsections.

#### 3.2.1. Bayesian Network 

In order to build Bayesian networks, it is first necessary to establish search mechanisms that can generate the DAG (Directed Acyclic Graph) using a set of heuristics that can reduce the number of combinations and generate the final Bayesian network. There are various Bayesian network search mechanisms, including tabu search [22], conditional independence [26], K2 [22], HillClimber [22], and TAN (Tree Augmented Naive Bayes) [27]. 

*(1) Tabu Search*. Tabu search can perform heuristic searches to select the structure from the Bayesian network best suited to a specific problem. A Tabu search can reduce the search area but does not guarantee finding the optimal solution.  Algorithm 1 shows the algorithm used to calculate the graph for the Bayesian network. *V* is the set of variables, *v*_*i*_ is the variable *i* and corresponds to node *i*. *π*_*i*_ is the set of parents for node *v*_*i*_, tabuList is the queue that stores the most recent movements, and *e*(*v*_*i*_, *π*_*i*_) is the expression used to calculate the quality of node *v*_*i*_. The study presented by [22] shows some of the proposals for this function.

Once the algorithm is complete, it returns the set of parents for each of the children nodes. 

*(2) Conditional Independence Test*. This algorithm is based on the calculation of the conditional independence test for the variables to generate a DAG that can obtain the probability estimates. If the variables being studied are independent, it will not be possible to generate a Bayesian network with good results. During the first phase, a graph containing the relationships between the variables is built; a DAG is then generated based on the previous graph. It is therefore necessary to take into consideration the number of categories of variables so that the analyses of independence are significant.  Algorithm 2 shows the procedure for creating a Bayesian network established for the conditional independence algorithm [26]. The function test in Algorithm 2 applies the chi-square statistical test [8] when 80% of the expected counts from the contingency table are greater than 5. Otherwise, Fisher's exact test [28] is applied. No statistical tests were applied for nominal variables since the data were discretized for the study, and the variables were converted to qualitative ordinals. 

*(3) Mixture of Bayesian Networks*. This work carries out a mixture of experts to minimize both the classification process and the false positives. False positives are defined by the value *y* = 0. The objective function is established according to (2). In order to give greater weight to false negatives, the *K* parameter, which has a default value of 1, must be modified:

(2) fα1,…,αn=∑iα1x1i+⋯+αnxni−yi2+kα1x1i+⋯+αnxni−yi2yi,

where *α*_*i*_ defines the weight of the classifier *i*, *x*_*i*_ is the predicted value of the classifier *i*,  *y*_*i*_ is the real output value, and *k* is the weighted value of the false positives (default value 1).

In order to give the correct weight to the output of the classifiers, (3) must be considered:

(3) α1+⋯+αn=1,

(4) fα1,…,αn=∑iα1x1i+⋯+αnxni−yi2+kα1x1i+⋯+αnxni−yi2yis.t.  α1+⋯+αn=1.

To optimize the problem, the method of Lagrange multipliers, as defined by (5), is applied, whereby

(5) Lα1,…,αn,λ=∑iα1x1i+⋯+αnxni−yi2+kα1x1i+⋯+αnxni−yi2yi−λα1+⋯+αn−1.

The optimum value is calculated as in the following equations:

(6) $$\begin{array}{c} \frac{\partial L}{\partial \alpha_{1}}=0,\\ \vdots \\ \frac{\partial L}{\partial \alpha_{n}}=0,\\ \frac{\partial L}{\partial \lambda }=0. \end{array}$$

### 3.3. Revise and Retain

The revise phase is carried out by applying techniques that attempt to express the classifications performed by the Bayesian network. The explanation of the Bayesian network includes statistical techniques to extract relevant variables during the classification process: the chi-square [8], the Yates correction tests [29], the chi-square with the Monte Carlo simulation [30], and Fisher's exact test [28] are applied to select the variables of interest that characterize the various pathologies. It is important to note that in order for the expected frequency to be less than 5, the result may be incorrect; consequently, Yates correction would be applied in an attempt to mitigate this issue. The statistical results from chi-squared test are also provided, applying the Monte Carlo simulation to verify the results. Finally, an exact Fisher test is applied, which is the recommended method when the sample size is small and it is not possible to ensure that 80% of the data from a contingency table have a value greater than 5. Medical studies such as [31] use a process similar to the one presented for selecting variables that affect malformations; other biomedical studies include [28–30]. There are many alternatives for correcting data, such as that in [32].

## 4. Case Study and Results Obtained

A case study was designed using the data from the patient files at the Faculty of Odontology, Masters of Endodontics, at the Complutense University of Madrid. All patients received root canal treatments between September 2000 and May 2014. Among all the patients treated during this time, we selected 205 cases (205 failures) that satisfied the inclusion criteria and were interested in a follow-up appointment. Success of retreatment of root canal therapy is defined as no presence of radiographic and clinical symptoms in a period of five years after the treatment was performed; failure of retreatment of root canal therapy is defined as the presence of radiographic lesions around the tooth retreated and presence of signs such as pain, movility, fistula, and inflammation. The retreatments were reviewed every year.

None of the patients from the selected cases who came for a follow-up treatment refused to participate in the study. The selected 205 cases contained all the information needed to complete the 72 variables being considered with 105 failures in retreatment. Some of the 72 variables were recombined in categorical values because they were binaries while the others were removed, for example, address of the treatment and sender. Certain initial variables included a high number of categories, which resulted in their recodification to ensure that the final number of categories per variable had around 3 or 4 different values. The final list of variables is summarized and described in Table 1. The variable to predict is highlighted in bold. These variables take into account all information relevant to the patient: medical and dental history and habits. Data relative to the state of the tooth prior to treatment were also included: the evolution, the clinical technique used, and the posttreatment results.

We then analyzed the system explained in Figure 1. The system was tested with the 205 cases to predict the failures in retreatment depending on the variables shown in Table 1. In summary, the reuse phase was analysed according to different configurations and using the accuracy rate and the area under the ROC curve (AUC); the relevant variables extracted in the revise phase are shown in this section.

In the retrieve phase, the system extracted the cases with retreatment and used them to generate the classifiers during the reuse phase, The system was compared with different classifiers applied in the reuse phase; those specifically applied include BayesNet, NaiveBayes, AdaBoostM1, Bagging, DecisionStump, J48, IBK, JRip, LMT, Logistic, LogitBoost, OneR, SMO, and Stacking. The results obtained by applying the leave-one-out technique are shown in Table 2. In summary, the test was carried out as follows: we extracted a case in the memory and then proceeded with the CBR system explained in Figure 1. We can observe that the accuracy rate of the system is greater than the other classifiers, although the procedure is insufficient to determine whether the differences are statistically significant.

To evaluate the significance of the different techniques presented in Table 2, a cross-validation was established following Dietterich's 5 × 2-cross-validation paired *t*-test algorithm [33]. Instead of using the accuracy rate, the AUC was used, since the classification problem is not symmetrical. Value 5 in the algorithm represents the number of replications of the training process, and value 2 is the number of sets into which the global set is divided (2-fold). Thus, for each technique, the global dataset *S* was divided into two groups *S*_1_ and *S*_2_ as follows: *S* = *S*_1_ ∪ *S*_2_ and *S*_1_∩*S*_2_ = *ϕ*. The learning and estimation stages were then carried out. This process was repeated 5 times for each technique and included the following steps: the classifier was trained using *S*_2_ and was then used to classify *S*_2_ and *S*_1_. In a second step, the classifier was trained using *S*_1_ and was then used to classify *S*_2_ and *S*_1_. The results obtained are shown in Table 3, where the columns represent the success rate obtained for *S*_1_, *S*_2_ (*R*_*i*_-A trained with *S*_1_) and *S*_1_, *S*_2_ (*R*_*i*_-B trained with *S*_2_) for each *i* repetition. The rows of Table 3 show the different classifiers previously shown in Table 2.

**Figure 2:**
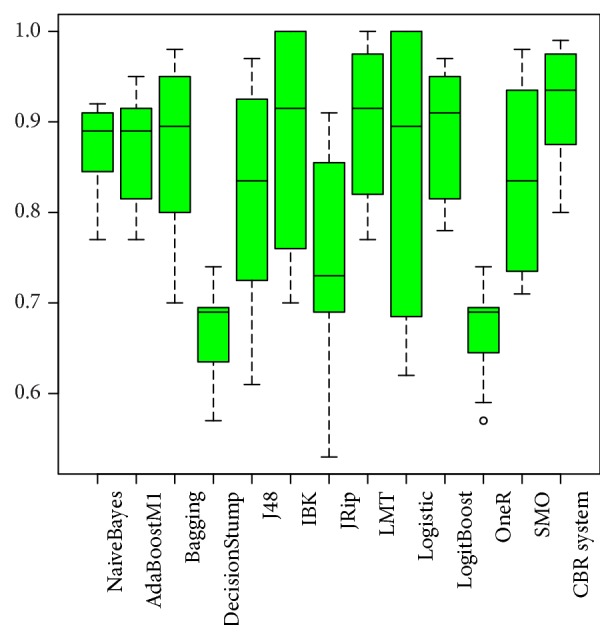
Distribution of the classifier values for each class.

Figure 2 shows the box plot associated with the AUC for each of the methods. As shown, the interquartile range for the CBR system is less than that for the other methods.

Once the results presented in Table 3 were obtained, a study on the significance of the different classification techniques was performed by applying the Mann-Whitney *U* test. It was a nonparametric test in which it is not necessary to make assumptions on the data distribution, as in the *t*-test. The test determines two values: *H*_0_ and *H*_1_. *H*_0_ shows whether the AUC value of the column classifier is greater than that of the file method, whereas *H*_1_ determines if the AUC for the ROC curve of the column is lower than the row. The values above the diagonal in Table 4 show the level of significance for the statistical test; therefore, if the column classifier has an area under curve greater than the file (i.e., level of significance > 0.05) it is shown in normal type; otherwise, it is shown in bold type. Clearly, the CBR system approach has a greater AUC for the ROC curve than the other methods.

The analysis of the cross-validation is completed using the Dietterich's 5 × 2-cross-validation paired *t*-test [33]. The results obtained are shown in the lower diagonal of Table 4. It is possible to observe that the results are very similar to those previously shown in the upper diagonal (Mann-Whitney *U* test). The values below the diagonal contain the results. In this case, the file classifier is compared to the column using the same hypotheses as the Mann-Whitney case. The values greater than 0.05 indicate that the area of the row classifier is greater than that of the column. The CBR system provides the best results in the test.

The mixture was compared to other Bayesian network search algorithms in order to analyze its results. As with the comparisons of other methods, a 5 × 2 cross-validation was performed, which provided the values shown in Table 5 for the AUC of the ROC curve. In this case, the average value appears to be less than the value for the methods shown in Table 3. The mixture increases the AUC provided for the other methods. By applying statistical tests as with Table 2, we can conclude that the value of the AUC for the mixture is statistically different for all methods with a significance level of 0.1. This was to be expected since the mixture is composed of both methods.

In order to explain the relevant variables during the reuse phase in the CBR system, the difference between the values of the variables for the categories of successful retreatments and extractions were analysed in the revise phase. To perform this analysis, the chi-square, Yates correction, chi-square with Monte Carlo simulation, and Fisher's exact tests were applied.  Table 6 displays the set of variables that were considered relevant by any of the three methods. We can see how the selection of variables coincides to a great degree for the different methods. This method for extracting relevant variables makes it possible to determine, to a large extent, the relevance of the variables that the Bayesian networks will have, particularly since the analysis of the dependence between variables is also used.

## 5. Conclusions

This paper has presented an innovative system specially designed to help odontologists make decisions about retreatment. The medical staff that participated in the experiments have remarked on the usefulness of the proposed approach and have noted that the system can be very helpful for their work.

The results obtained show that, with the CBR analysis, the data obtained were relevant because by ordering the established variables, particularly those with the highest risk factor, we could predict the final solution for treatment and retreatment in 84.4% of the cases, by applying the leave-one-out techniques.

The combination applied in the mixture increases the AUC for the ROC curve, thus increasing the rate of accuracy for the results, which is important when working with nonsymmetrical case studies. The mixture also makes it possible to reduce the number of false negatives by placing great importance on the possibility of false positives. Moreover, the objective function can be modified depending on the case study, thus allowing for an increase in the relevance of some metrics.

In other case studies, it could be necessary to analyze the classifiers in the mixture in order to optimize the objective functions. In this case, the mixture of the Bayesian networks provided good results in some cases, while in others we could use alternative techniques such as decision trees in order to provide some rules to explain the classification in the revise phase.

Furthermore, the system makes it possible to extract the relevant variables that can distinguish the different types of retreatments. Nevertheless, more cases are required to contrast the results with greater accuracy.

The system can reduce the number of unsuccessful retreatments because it predicts the rate of success or failure, thus avoiding unnecessary extractions.

## Figures and Tables

**Algorithm 1:**
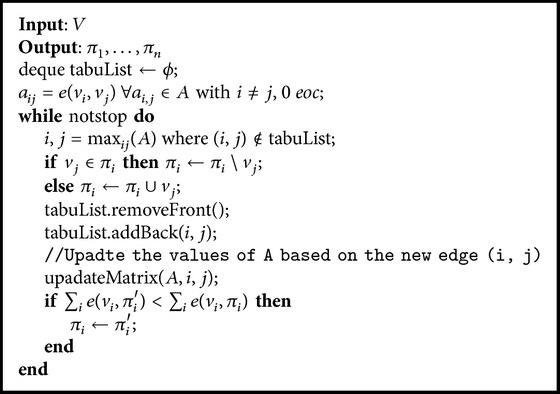
Generation of a Bayesian network using the tabu search algorithm.

**Algorithm 2:**
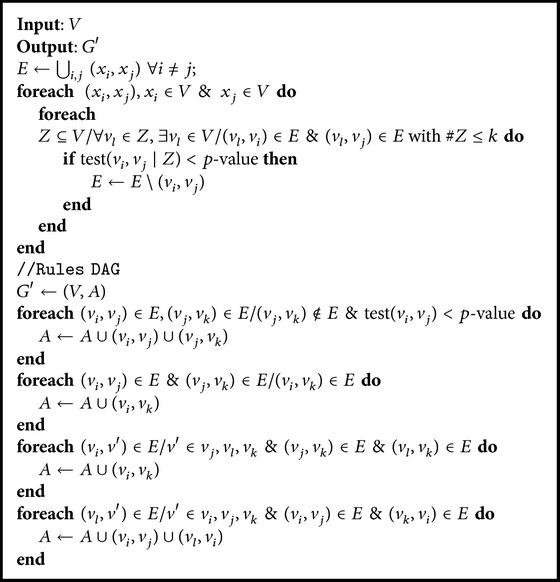
Condition independence.

**Table 1:** Description of the final preprocessed variables.

Variable	Class
Habits-parafunctions	Categorical 2 values
General pathology	Binary
Total current treatments	Binary
Allergy	Binary
Sessions	Discrete
Mechanical/manual instrumentation	Binary
Lateral or vertical	Binary
Anesthetic	Categorical 3 values
Clamps	Categorical 4 values
Ranking difficulty level	Categorical 3 values
Student course	Discrete 4 values
Tooth position	Categorical 3 values
Anatomical characteristics of the crown	Categorical 3 values
Root anatomy	Categorical 3 values
Anomalies	Binary
Type of restoration: Perno	Binary
Perno	Binary
Type	Binary
Diámetro diameter	Categorical 5 values
Length	Categorical 3 values
Time endodontics-restoration	Categorical 4 values
Type of pain	Categorical 4 values
Inflammation	Binary
Fistula	Binary
Number of roots	Discrete 3 values
Number of tubes	Discrete 4 values
Root morphology	Binary
Curvatures	Binary
Degree	Categorical 3 values
Bone level	Categorical 3 values
Stable occlusion	Categorical 3 values
Fracture type	Categorical 2 values
Location	Categorical 5 values
Signs of fissure/fracture	Binary
Probing	Binary
Movility	Binary
visible crack	Binary
Level	Binary
Time to failure	Categorical 4 values
Retreatments	Binary
Percha solvent	Binary
Use of rotary	Binary
**Failures in retreatment**	**Binary**

**Table 2:** Correct classifications.

Classifier	Correct
CBR system with Bayesian networks	173
NaiveBayes	157
AdaBoostM1	162
Bagging	149
DecisionStump	141
J48	154
IBK	154
JRip	158
LMT	161
Logistic	152
LogitBoost	168
OneR	141
SMO	163

**Table 3:** AUC for the different classifiers applying 5 × 2 cross-validation.

	*R*_1_-A		*R*_1_-B		*R*_2_-A		*R*_2_-B		*R*_3_-A		*R*_3_-B		*R*_4_-A		*R*_4_-B		*R*_5_-A		*R*_5_-B		Average test samples
	*S*_1_	*S*_2_	*S*_1_	*S*_2_	*S*_1_	*S*_2_	*S*_1_	*S*_2_	*S*_1_	*S*_2_	*S*_1_	*S*_2_	*S*_1_	*S*_2_	*S*_1_	*S*_2_	*S*_1_	*S*_2_	*S*_1_	*S*_2_	
NaiveBayes	0.90	0.89	0.91	0.84	0.92	0.83	0.90	0.77	0.92	0.79	0.92	0.89	0.92	0.85	0.89	0.86	0.91	0.84	0.90	0.88	0.84
AdaBoostM1	0.89	0.89	0.91	0.81	0.93	0.77	0.91	0.78	0.95	0.82	0.90	0.82	0.93	0.79	0.91	0.87	0.92	0.82	0.92	0.81	0.82
Bagging	0.95	0.87	0.95	0.84	0.97	0.70	0.96	0.73	0.98	0.80	0.94	0.80	0.92	0.75	0.94	0.83	0.97	0.80	0.92	0.73	0.79
DecisionStump	0.69	0.69	0.64	0.57	0.69	0.57	0.74	0.64	0.69	0.59	0.73	0.65	0.72	0.63	0.69	0.69	0.70	0.68	0.72	0.63	0.63
J48	0.92	0.79	0.96	0.79	0.91	0.76	0.93	0.73	0.97	0.70	0.91	0.77	0.97	0.69	0.88	0.70	0.94	0.61	0.92	0.72	0.73
IBk	1.00	0.77	1.00	0.78	1.00	0.74	1.00	0.70	1.00	0.71	1.00	0.83	1.00	0.79	1.00	0.82	1.00	0.75	1.00	0.74	0.76
JRip	0.79	0.67	0.80	0.69	0.76	0.53	0.90	0.62	0.86	0.67	0.91	0.71	0.90	0.70	0.69	0.69	0.89	0.72	0.85	0.74	0.68
LMT	1.00	0.85	0.97	0.81	0.94	0.77	0.98	0.81	0.99	0.79	0.96	0.83	0.98	0.80	0.94	0.89	1.00	0.83	0.96	0.83	0.82
Logistic	1.00	0.70	1.00	0.64	1.00	0.65	1.00	0.69	1.00	0.62	1.00	0.62	1.00	0.71	1.00	0.68	1.00	0.73	1.00	0.79	0.68
LogitBoost	0.93	0.90	0.95	0.82	0.96	0.78	0.95	0.81	0.97	0.81	0.94	0.86	0.95	0.78	0.92	0.89	0.95	0.80	0.94	0.83	0.83
OneR	0.69	0.69	0.69	0.66	0.69	0.57	0.74	0.64	0.69	0.59	0.73	0.65	0.72	0.63	0.69	0.69	0.70	0.68	0.72	0.63	0.64
SMO	0.92	0.77	0.95	0.73	0.96	0.72	0.91	0.72	0.98	0.72	0.91	0.75	0.96	0.75	0.91	0.75	0.97	0.74	0.90	0.71	0.74
CBR system	0.97	0.89	0.96	0.86	0.98	0.86	0.97	0.81	0.99	0.80	0.97	0.90	0.98	0.88	0.97	0.91	0.98	0.90	0.98	0.87	0.87

**Table 4:** Mann-Whitney and paired *t*-test for the significance of the differences. The upper diagonal contains the Mann-Whitney *U* test. The values greater than 0.05 indicate that the file classifier has an AUC bigger than the row classifier. The lower diagonal contains the *t*-test; the values greater than 0.05 indicate that the row classifier has an AUC greater than the column classifier.

	NaiveBayes	AdaBoostM1	Bagging	DecisionStump	J48	IBk	JRip	LMT	Logistic	LogitBoost	OneR	SMO	CBR system
NaiveBayes		0.05	**0.01**	**0.00**	**0.00**	**0.00**	**0.00**	0.05	**0.00**	0.18	**0.00**	**0.00**	0.96
AdaBoostM1	0.06		0.12	**0.00**	**0.00**	**0.01**	**0.00**	0.63	**0.00**	0.74	**0.00**	**0.00**	0.99
Bagging	**0.01**	0.08		**0.00**	**0.01**	0.18	**0.00**	0.94	**0.00**	0.94	**0.00**	**0.02**	1.00
DecisionStump	**0.00**	**0.00**	**0.00**		1.00	1.00	0.98	1.00	0.98	1.00	0.70	1.00	1.00
J48	**0.00**	**0.00**	**0.01**	1.00		0.94	**0.03**	1.00	**0.04**	1.00	**0.00**	0.60	1.00
IBk	**0.00**	**0.00**	0.16	1.00	0.95		**0.00**	1.00	**0.00**	1.00	**0.00**	0.06	1.00
JRip	**0.00**	**0.00**	**0.00**	0.95	**0.03**	**0.00**		1.00	0.46	1.00	**0.03**	1.00	1.00
LMT	0.09	0.58	0.94	1.00	1.00	1.00	1.00		**0.00**	0.57	**0.00**	**0.00**	0.99
Logistic	**0.00**	**0.00**	**0.00**	0.98	**0.04**	**0.00**	0.60	**0.00**		1.00	0.05	1.00	1.00
LogitBoost	0.17	0.70	0.96	1.00	1.00	1.00	1.00	0.64	1.00		**0.00**	**0.00**	0.98
OneR	**0.00**	**0.00**	**0.00**	0.69	**0.00**	**0.00**	0.08	**0.00**	**0.04**	**0.00**		1.00	1.00
SMO	**0.00**	**0.00**	**0.01**	1.00	0.69	**0.04**	0.99	**0.00**	0.99	**0.00**	1.00		1.00
CBR system	0.91	1.00	1.00	1.00	1.00	1.00	1.00	1.00	1.00	0.98	1.00	1.00	

**Table 5:** AUC of the different classifiers.

	*R*_1_-A		*R*_1_-B		*R*_2_-A		*R*_2_-B		*R*_3_-A		*R*_3_-B		*R*_4_-A		*R*_4_-B		*R*_5_-A		*R*_5_-B		Average test samples
	*S*_1_	*S*_2_	*S*_1_	*S*_2_	*S*_1_	*S*_2_	*S*_1_	*S*_2_	*S*_1_	*S*_2_	*S*_1_	*S*_2_	*S*_1_	*S*_2_	*S*_1_	*S*_2_	*S*_1_	*S*_2_	*S*_1_	*S*_2_	
Global HillClimber	1.00	0.73	1.00	0.84	1.00	0.84	1.00	0.79	1.00	0.72	1.00	0.76	1.00	0.83	1.00	0.78	1.00	0.85	1.00	0.84	0.80
Global TabuSearch	0.99	0.79	0.98	0.85	0.99	0.82	0.99	0.85	0.98	0.86	0.99	0.79	0.98	0.86	0.99	0.85	0.99	0.82	0.98	0.87	0.84
Global K2	1.00	0.81	1.00	0.80	1.00	0.83	1.00	0.89	1.00	0.84	1.00	0.82	1.00	0.85	1.00	0.82	1.00	0.86	1.00	0.85	0.84
Global TAN	0.99	0.78	0.99	0.83	1.00	0.87	0.99	0.88	0.99	0.82	0.99	0.81	0.99	0.85	1.00	0.82	0.99	0.85	1.00	0.86	0.84
CISearchAlgorithm	0.93	0.83	0.90	0.79	0.92	0.84	0.92	0.83	0.90	0.87	0.92	0.82	0.90	0.80	0.93	0.85	0.90	0.82	0.92	0.84	0.83
Local TabuSearch	0.97	0.83	0.96	0.83	0.96	0.88	0.97	0.87	0.96	0.86	0.97	0.84	0.97	0.83	0.97	0.87	0.97	0.85	0.98	0.83	0.84
Local K2	0.99	0.83	1.00	0.79	0.99	0.87	1.00	0.86	1.00	0.80	1.00	0.82	1.00	0.89	1.00	0.82	1.00	0.81	1.00	0.87	0.84
Local TAN	0.98	0.84	0.99	0.83	0.98	0.86	0.99	0.86	0.99	0.82	1.00	0.81	0.98	0.86	1.00	0.86	0.99	0.85	0.99	0.87	0.84
Mixture Local TabuSearch CISearchAlgorithm	0.99	0.85	1.00	0.83	0.99	0.88	1.00	0.90	0.99	0.86	0.99	0.84	0.99	0.90	0.99	0.88	1.00	0.84	1.00	0.87	0.87

**Table 6:** Relevant variables.

Variable	*p* value		
	Chi-squared test		Exact Fisher test
	Yates	Monte Carlo	
Allergy	0.01908788	0.02098951	0.01739722
Mechanical/manual instrumentation	0.0035934	0.001999	0.0029985
Anesthetic	0.00306269	0.0029985	0.00149925
Ranking difficulty level	2.33*E* − 06	0.00049975	0.00049975
Tooth number	0.00465514	0.00649675	0.00449775
Root anatomy	3.39*E* − 07	0.00049975	0.00049975
Type of restoration: Perno	6.28*E* − 06	0.00049975	0.00049975
Perno	0.00017239	0.00049975	9.61*E* − 05
Type	0.00537458	0.003998	0.00385604
Time endodontics-restoration	3.74*E* − 07	0.00049975	0.00049975
Length	0.00601985	0.00549725	0.00549725
Number of roots	0.02352122	0.02598701	0.02198901
Curvatures yes/no	0.02317278	0.02998501	0.02240645
Adjacent remaining	0.00109389	0.0029985	0.00149925
